# Candidate microRNA Biomarkers in Human Gastric Cancer: A Systematic Review and Validation Study

**DOI:** 10.1371/journal.pone.0073683

**Published:** 2013-09-09

**Authors:** Ji-Lin Wang, Ye Hu, Xuan Kong, Zhen-Hua Wang, Hao-Yan Chen, Jie Xu, Jing-Yuan Fang

**Affiliations:** 1 Division of Gastroenterology and Hepatology, Renji Hospital, Shanghai Jiao-Tong University School of Medicine, Shanghai Institution of Digestive Disease, Shanghai, China; 2 Key Laboratory of Gastroenterology & Hepatology, Ministry of Health, Shanghai, China; 3 State Key Laboratory of Oncogene and Related Genes, Shanghai, China; Queen Elizabeth Hospital, Hong Kong

## Abstract

Gastric cancer (GC) remains a major cause of morbidity and mortality worldwide and there is therefore a clear need to search for more sensitive early diagnostic biomarkers. We performed a systematic review of eight published miRNA profiling studies that compared GC tissues with adjacent noncancerous tissues. A miRNA ranking system was used that took the frequency of comparisons, direction of differential expression and total sample size into consideration. We identified five miRNAs that were most consistently reported to be upregulated (miR-21, miR-106b, miR-17, miR-18a and miR-20a) and two miRNAs that were downregulated (miR-378 and miR-638). Six of these were further validated in 32 paired sets of GC and adjacent noncancerous tissue samples using real-time PCR. MiR-21, miR-106b, miR-17, miR-18a and miR-20a were confirmed to be upregulatedin GC tissues, while the expression of miR-378 was decreased. Moreover, we found a significant association between expression levels of miR-21, miR-106b, miR-17, miR-18a and miR-20a and clinicopathological features of GC. These miRNAs may be used for diagnostic and/or prognostic biomarkers for GC and therefore warrant further investigation.

## Introduction

Despite a recent decrease in the incidence of gastric cancer (GC) [1], it remains a cause of major morbidity and mortality worldwide, especially in Eastern Asia. A total of one million new cases of GC occurred in 2008, with 738,000 deaths [2]. This accounts for 8% of the total cases of cancer and 10% of total deaths. Although endoscopy can detect the early stages of GC, most cases are still diagnosed at an advanced stage, which results in a poor prognosis [3]. The 5-year survival rate for GC cases with stage II ranges from 30% to 50%, but falls to between 10% and 25% for patients with stage III disease [4]. Although endoscopic techniques are developing rapidly, their value for the early detection of GC is limited due to a lack of sensitivity, high costs and inconvenience. New diagnostic and prognostic biomarkers for GC are therefore urgently required.

MicroRNAs (miRNA) are short noncoding RNA molecules of 19–25 nt. They regulate gene expression at the post-translational level by guiding the RNA-induced silencing complex to miRNA target sites in the 3′ untranslated region of mRNA, leading to mRNA degradation or the inhibition of translation [5]. Previous studies have shown that numerous miRNAs are aberrantly expressed in many kinds of cancers, and miRNA expression profiling has shown certain miRNAs to be associated with tumor development, progression and response to therapy. They are therefore good candidates for using as diagnostic, prognostic and predictive biomarkers [6].

Several studies have been conducted to search for biomarkers by identifying the differential expression of miRNAs between GC tissue samples and corresponding non-tumor gastric tissue from the same patient [7]–[14]. These studies have resulted in the identification of hundreds of differentially expressed miRNAs. However, many of these are likely to be false positives, and only a small fraction could be used as diagnostic or prognostic biomarkers. A logical approach to distinguish important miRNAs from a large number of candidate miRNA lists is to search for the intersection of miRNAs identified in multiple independent studies [15]. Although this method has become increasing popular [15], [16], [17], no published study has identified the intersections of GC-related miRNAs based on a large number of miRNA expression profiling studies.

We conducted this systematic review to identify the most important differentially expressed miRNAs that have been consistently reported in a series of independent miRNA expression profiling studies in GC patients. Moreover, we further validated some of the miRNAs that were most up- or downregulated using real-time PCR in 32 pairs of GC and matched adjacent non-tumor tissue samples.

## Materials and Methods

### Ethics Statement

The study was approved by the ethics committee of Shanghai Jiaotong University School of Medicine, and written informed consent was obtained from all patients at study entry.

### Search Strategy

Potential studies published in English were collected from Medline using the following keywords: ‘miRNA’ OR ‘microRNA’ OR ‘miR’, ‘gastric’ OR ‘stomach’, ‘profiling’ OR ‘microarray’. Lists of references of review articles and original articles were searched manually for additional publications.

### Inclusion Criteria of the Literature

For a study to be included in this systematic review, several criteria had to be met: 1) studies had to be miRNA profiling studies in GC patients; 2) studies had to use GC tissues and their corresponding adjacent non-tumor tissues for comparison; 3) methods had to comprise miRNA microarray techniques. Furthermore, only full-text publications in English were included. The profiling studies that used GC cell lines or serum samples from GC patients, those that compared GC biopsies from tumors with different stages of disease, and those that used different miRNA technologies were not included. Review articles were also not included in this systemic review.

### Data Extraction and Lists of miRNA

Differentially expressed miRNAs were identified from each included profiling study. Relevant information was determined (i.e., chromosomal location, pre-miRNA length, mature miRNA sequence and potential targets of the miRNAs), and missing information was identified from the miRBase database (www. mirbase.org/) and Pubmed.

### Ranking

Each included profiling study [7]–[14] provided a list of differentially expressed miRNAs (Table S1). Griffith and Chan devised a method to rank potential molecular biomarkers for comparison groups [16], [17], which has been used for miRNA profiling studies. For example, Ma et al. [15] identified the intersections of colorectal cancer-related miRNAs based on a large number of miRNA profiling studies. Thus, the criteria for the literature included in this current systematic review were based on those in their reports [15]. MiRNAs were ranked to the criteria in the following order of importance: (i) the miRNA was consistently reported as differentially expressed in a consistent direction of change; (ii) the frequency of the miRNA was reported in the microarray studies; (iii)the total sample size for each consistent reported miRNAs.

### Validation of the miRNAs Using Real Time PCR

To validate the profiling results, 32 fresh GC tissues and their paired non-tumor gastric tissues were obtained from the Renji Hospital, affiliated to the Shanghai Jiaotong University School of Medicine. Total RNA was extracted from 32 pairs of matched human GC specimens (including cancer and adjacent noncancerous tissues) using TRIzol reagent (Invitrogen). The RNA concentration and purity was measured using Nanodrop ND-2000, and the ultraviolet absorption measurement method was applied to detect the purity of the RNA, only those A260/A280 located between 1.80–2.00, and A260/A230>1.7, were used for the final experiment, otherwise the RNA must be re- extracted. Reverse transcription from 3 µg RNA was done usingAll-in-OneTM First-Strand cDNA Synthesis Kit(Genecopoeia, Guangzhou, China), according to the manufacturer’s protocol. In brief, the prepared RNA reverse transcription reaction solution was incubated at 37°C for 60 minutes and terminated at 85°C for 5 minutes, and then stored at −20°C for further analysis. Quantitative PCR (qPCR) was performed using an ABI Prism 7900HT Sequence Detection System (Applied Biosystems, USA) with SYBR Premix Ex Taq II (Takara). The primers (miR-21-5p, miR-106b-5p, miR-17-5p, miR-18a-5p, miR-20a-5p and miR-378-5p) including U6 were obtained from Genecopoeia (Guangzhou, China). Quantification was calculated using the 2 −ΔΔCT method and is presented as normalized pattern.

### Statistical Analysis

The results were analyzed using SAS 9.2 software (SAS Institute Inc. USA). Data are presented as means ± SD. Student’s t-test was used to compare values between two independent groups.

## Results

### Literature Selection and Study Characteristics

**Figure 1:**
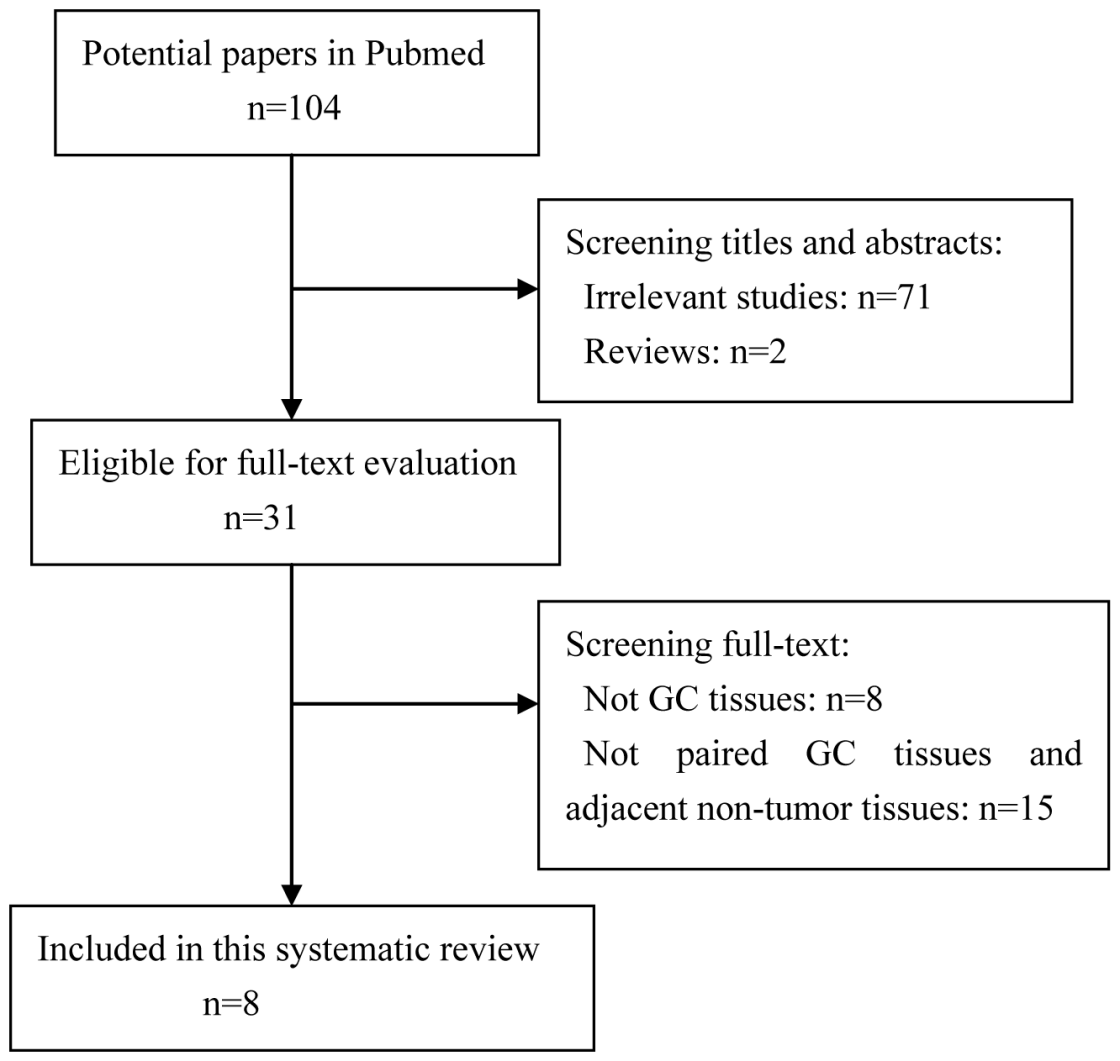
Flowchart of the study selection.

A total of 104 studies were searched in Pubmed using our search strategy, 73 of which were excluded after screening the titles and abstracts. 23 studies were excluded after reading the full text. Only eight studies were finally included in this systematic review. The detailed study selection was shown in Figure 1. The detailed characteristics of each study are given in Table 1.

**Table 1:** The characterestics of the eight microRNA profiling studies included in this systemic review.

Study	Year	Platform	Total samplesize	No. of differentialmiRNAs	Up-regulatedmiRNAs in GC	Down-regulatedmiRNAs in GC
Saito et al [7]	2012	Toray Industries	20	10	NR	10
Wang et al [8]	2012	miRCURY LNA microarray platform	6	18	NR	18
		(Exiqon, Denmark)				
Oh et al [9]	2011	Agilent Human miRNA microarray(V2)	80	80	40	40
Ding et al [10]	2010	µParaflo™ microfluidic chip	12	15	8	7
		(LC Sciences, TX, USA)				
Tchernitsa et al [11]	2010	NCODE muti-species microarray probe set	12	22	20	2
		(Invitrogen, CA, USA)				
Ueda et al [12]	2010	Ohio State University custom microRNA	320	35	22	13
		microarray chip				
Yao et al [13]	2009	miRCURY LNA array (V 11.0)	6	24	22	2
Guo et al [14]	2008	µParaflo™ microfluidic chip	6	19	12	7
		(LC Sciences, TX, USA)				

### Differentially Expressed miRNAs

A total of 223 differentially expressed miRNAs were reported in the eight microarray studies (Differentially expressed miRNAs in each study were detailed in Table S1); 124 were upregulated in GC, and 99 were downregulated. Among the 223 differentially expressed miRNAs, 48 were reported in at least two studies; 39 (81.3%) had a consistent direction and nine (18.7%) had an inconsistent direction of altered expression. Among the former 39, 20 were upregulated in GC, and 19 were downregulated. Three of these miRNAs were reported in five microarray studies (miR-21, miR-106b and miR-378), four were reported in four studies (miR-17, miR-18a, miR-20a and miR-638), and seven were reported in three studies (miR-19a, miR-20b, miR-25, miR-30d, miR-923, miR-375, and miR-148a). Their chromosomal locations, pre-miRNA lengths, mature sequences and the potential targets are listed in Tables 2–4.

**Table 2:** Up-regulated miRNAs (n = 20) reported in at least two expression profiling studies.

miRNAsname	Chromosomallocalization	Pre-miRNAlength	Mature sequence	Studies(reference)	Total samplesizes	Potential Target
miR-21	17q23.1	72	8-uagcuuaucagacugauguuga - 29	5 [9], [10], [11], [12], [14]	430	PTEN, VEGF,STAT3,MMP-2
miR-106b	7q22.1	81	13-aaaagugcuuacagugcagguag-35	5 [10], [11], [12], [13], [14]	356	RB1, CDKN1A,IL-10,RUNX1
miR-17	13q31.3	71	14-caaagugcuuacagugcagguag-36	4 [9], [12], [13], [14]	412	STAT3,ZNFX1,EIF5A2,SMAD12
miR-18a	13q31.3	71	6-uaaggugcaucuagugcagauag-28	4 [9], [12], [13], [14]	412	DICER1,Myc,HIF1A,Eralpha
miR-20a	13q31.3	71	8-uaaagugcuuauagugcagguag-30	4 [9], [10], [12], [14]	418	SERF1A,PKD2,SMAD12,TRPV6
miR-19a	13q31.3	71	14-aguuuugcauaguugcacuaca-35	3 [9], [12], [14]	406	TNF-α,SOCS1,CCND1,TGFBR2
miR-20b	Xq26.2	71	6-caaagugcucauagugcagguag-28	3 [10], [12], [14]	338	CDKN1A,MYLIP,ESR1,STAT3
miR-25	7q22.1	84	14-aggcggagacuugggcaauug-34	3 [10], [11], [12]	344	F8A3,FAM47C,FBXO21,LY9
miR-18b	Xq26.2	71	6-uaaggugcaucuagugcaguuag-28	2 [9], [14]	86	Eralpha, ESR1
miR-340	5q35.3	95	16-uuauaaagcaaugagacugauu-37	2 [13], [14]	12	PELI1,PTPDC1,RNF11,SOX4
miR-214	1q24.3	110	30-ugccugucuacacuugcugugc -51	2 [9], [11]	92	BUB3,RFX3,KLF12,ARHGAP12
miR-224	Xq28	81	8-caagucacuagugguuccguu-28	2 [9], [12]	400	API5L1,RAB9B,CXCR4,CD40
miR-135b	1q32.1	97	16-uauggcuuuucauuccuauguga-38	2 [9], [12]	400	BGLAP,APC,RUNX2,KLF4
miR-34a	1p36.22	110	22-uggcagugucuuagcugguugu-43	2 [9], [13]	86	SIRT1,MYC,HDAC1,LEF1
miR-125b	11q24.1	88	15-ucccugagacccuaacuuguga-36	2 [9], [11]	92	p53,MUC1,VDR,IL-6
miR-27a	19p13.13	78	10-agggcuuagcugcuugugagca-31	2 [9], [11]	92	SGOL1,ZNF135,NPM1,DLEU1
miR-103	5q34	78	48-agcagcauuguacagggcuauga-70	2 [9], [11]	92	DICER1,AXIN2,HRB,ARIH2
miR-223	Xq12	110	26-cguguauuugacaagcugaguu-47	2 [9], [13]	86	GABRB3,SEMA3A,TRAPPC10
miR-181a-2	9q33.3	110	39-aacauucaacgcugucggugagu-61	2 [12], [13]	326	p27,CDX2,BCL2,K-RAS,GATA6
miR-92	13q31.3	78	11-agguugggaucgguugcaaugcu-33	2 [11], [12]	332	PARP2,CXCL9,SIX3,NRP2

**Table 3:** Down-regulated miRNAs (n = 20) reported in at least two expression profiling studies.

miRNAsname	Chromosomallocalization	Pre-miRNAlength	Mature sequence	Studies(reference)	Total samplesizes	Potential Target
miR-378	5q32	66	5 -cuccugacuccagguccugugu-26	5 [7], [8], [9], [13], [14]	118	CDK6, VEGF,POLH,DFFA
miR-638	19p13.2	100	16-agggaucgcgggcggguggcggccu-40	4 [8], [9], [10], [13]	104	NKX2,CCNG2,WDR47,GPR116
miR-30d	8q24.22	70	6 -uguaaacauccccgacuggaag-27	3 [9], [10], [12]	412	RUNX2, TP53,GNAI2
miR-923*				3 [7], [9], [10]	112	
miR-375	2q35	64	40 -uuuguucguucggcucgcguga-61	3 [9], [10], [12]	412	JAK2,HuD, N-Cadherin,CASP3
miR-148a	7p15.2	68	6 -aaaguucugagacacuccgacu-27	3 [9], [11], [12]	412	POU2F1,BRWD1,VASH2,GSR
miR-31	9p21.3	70	8 -aggcaagaugcuggcauagcu- 28	2 [8], [14]	12	RHOA,FOXP3,ARPC5,CASR
miR-133b	6p12.2	119	66-uuugguccccuucaaccagcu -87	2 [7], [14]	26	CASP9,BCL2L2,IGF1R,PITX3
miR-139	11q13.4	68	7-ucuacagugcacgugucuccagu-29	2 [9], [14]	86	TMF1,FMR1,EIF4,EBF1
miR-768*				2 [7], [14]	26	
miR-141	12p13.31	95	17- caucuuccaguacaguguugga-38	2 [8], [10]	18	CD46,EGFR,DLC1,MYOCD
miR-663	17q23.2	93	15-aggcggggcgccgcgggaccgc -36	2 [8], [10]	18	ZFR2,GRIN2D,PRRT1,SHOX
miR-494	14q32.31	81	48-ugaaacauacacgggaaaccuc -69	2 [8], [9]	88	PTEN,CDK6,
miR-623	13q32.3	98	16- aucccuugcaggggcuguugggu-38	2 [8], [9]	88	IL6ST,AR,ZNF197,ACSM2A
miR-30c	6q13	70	17-uguaaacauccuacacucucagc -39	2 [10], [12]	332	MUC17,UBE2I
miR-193b	16p13.12	83	14 -cgggguuuugagggcgagauga -35	2 [7], [9]	100	ARL8B,CACS3,PCDH17,CCR1
miR-939	8q24.3	82	15 -uggggagcugaggcucugggggug-38	2 [7], [9]	100	IL-6, TNF-α
miR-29c	1q32.2	88	16 -ugaccgauuucuccugguguuc-37	2 [7], [12]	340	TCEA3,PPP2R5E,HEBP2,MAFA
miR-30a	6q13	70	6 -uguaaacauccucgacuggaag-27	2 [9], [12]	400	RUNX2,MET,CDH1,WNT5A

* Human mir-923 appears to be a frgament of the 28S rRNA, so is removed from the microRNA database;

Human mir-768 overlaps an annotated snoRNA, HBII-239. Phylogenetic analysis in all vertebrates supports the snoRNA annotation, with poor conservation of the reported mature miRNA sequence,it is therefore removed from the microRNA database.

**Table 4:** The differentially expressed miRNAs (n = 9) with an inconsistent direction between studies.

miRNAsname	Chromosomallocalization	Pre-miRNAlength	Mature sequence	Studies(reference)	Direction ofexpression	Totalsamplesizes	Potential Target
miR-106a	Xq26.2	70	13-aaaagugcuuacagugcagguag-35	14	↑	6	RB1, CDKN1A, E2F1, VEGFA
				9	↓	80	
miR-27a	19p13.13	70	10 -agggcuuagcugcuugugagca-31	9	↑	80	CUX1, SMAD12, PIGK, PCDHB4
				8	↓	6	
miR-107	10q23.31	70	50 -agcagcauuguacagggcuauca -72	9	↑	80	PLAG1, CDK6, BACE1, CRKL
				8	↓	6	
miR-200b	1p36.33	94	21-caucuuacugggcagcauugga-42	9	↑	80	ZNF396, RAB22A, FZD1, FLRT3
				8	↓	6	
miR-181c	19p13.13	70	27 -aacauucaaccugucggugagu-48	12	↑	320	IL-2, BCL-2, CDX2, GATA6
				9	↓	80	
miR-345	14q32.2	98	18 -gcugacuccuaguccagggcuc-39	12	↑	320	ABCC1, NTRK3, CDKN1A
				9	↓	80	
miR-29b	7q32.3	80	10-gcugguuucauauggugguuuaga-33	12	↑	320	USP28, LIN9, WDR26, PROS1
				9	↓	80	
miR-451	17q11.2	72	17-aaaccguuaccauuacugaguu-38	12	↑	320	CXCL16, CDKN2B, PSMB8, MBP
				9	↓	80	
miR-222	Xp11.3	110	31-cucaguagccaguguagauccu-52	11	↑	12	PTPN3, BCAR3, ADAM22,CD2AP
				9	↓	80	

### Validation of the Selected miRNAs in GC Patients

**Figure 2:**
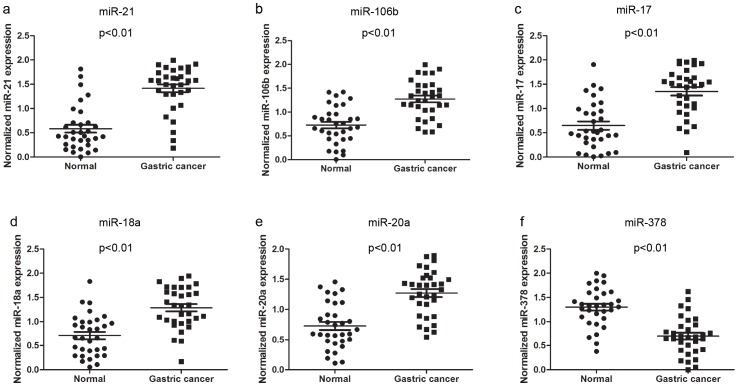
Expression levels of miR-21, miR-106b, miR-17, miR-18a, miR-20a and miR-378 in GC and adjacent noncancerous tissue samples. Using U6 as a normalization control, the expression of miR-21, miR-106b, miR-17, miR-18a and miR-20a was significantly higher in GC tissues, while the expression of miR-378 was significantly lower.

To validate the expression of the six most consistently reported miRNAs (miR-21, miR-106b, miR-17, miR-18a, miR-20a and miR-378), the expression of these miRNAs in GC biopsies and adjacent noncancerous tissues were compared in 32 cases of GC using real-time PCR. The raw Ct values of the six miRNAs were shown in Table S2.The results showed that miR-378 was downregulated in GC tissues, whereas the other five miRNAs (miR-21, miR-106b, miR-17, miR-18a and miR-20a) were upregulated in GC (Figure 2). Our results were consistent with those of the original profiling studies. Furthermore, we explored the relationship between the expression of these miRNAs with the clinical and pathological features of GC. We found that the expression of miR-21 was significantly higher in cases of GC cases with larger tumor sizes (≥8 cm), poor differentiation and metastasis with lymph node involvement and later stage disease. MiR-106b, miR-17 and miR-18a levels were significantly higher in poorly differentiated GC, cases with lymph node involvement, or late stage disease, while miR-20a levels were significantly higher in cases of GC with lymph node involvement. However, no relationship was found between the expression of miR-378 and the clinicopathological features of GC. These results are detailed in Table 5.

**Table 5:** The relationship between the six selected microRNAs and gastric cancer characteristics.

	miR-21		miR-106b		miR-17		miR-18a		miR-20a		miR-378	
	ΔCt	p value	ΔCt	p value	ΔCt	p value	ΔCt	p value	ΔCt	p value	ΔCt	p value
**Tumor size**												
<8 cm	−12.07±0.62	0.02	−1.56±0.40	0.48	−4.67±0.70	0.48	0.99±0.43	0.01	−4.46±0.59	0.41	4.06±0.31	0.84
≥8 cm	−14.32±0.63		−1.94±0.33		−4.07±0.39		−0.48±0.34		−5.09±0.47		3.85±0.75	
**Location**												
Cardia	−13.61±0.66	0.57	−0.97±0.50	0.18	−2.68±0.61	0.04	0.80±0.72	0.47	−4.11±0.66	0.42	5.22±1.01	0.23
Nocardia	−12.89±0.57		−1.90±0.29		−4.77±0.45		0.21±0.34		−4.92±0.43		3.66±0.57	
**Lauren’s classification**												
Diffuse	−13.37±0.70	0.43	−1.91±0.36	0.45	−4.64±0.50	0.49	0.11±0.43	0.47	−4.83±0.38	0.86	3.89±0.70	0.58
Intestinal	−12.61±0.65		−1.50±0.38		−4.09±0.67		0.57±0.46		−4.69±0.71		4.06±0.77	
**Differentiation**												
Well	−11.68±0.68	0.02	−0.84±0.42	0.01	−3.15±0.56	0.03	1.29±0.38	0.02	−4.12±0.61	0.27	4.78±0.87	0.28
Poor	−13.89±0.59		−2.18±0.29		−5.01±0.49		−0.16±0.39		−5.05±0.47		3.63±0.57	
**Metastasis with lymph node**												
No	−11.18±1.00	0.02	−0.80±0.19	0.04	−5.37±1.30	<0.001	1.14±0.15	0.17	−3.17±0.60	0.03	5.62±0.98	0.07
Yes	−13.72±0.48		−2.03±0.25		0.10±0.31		0.11±0.31		−5.21±0.40		3.29±0.55	
**pTNM Stage**												
Early(I+II)	−11.62±0.75	0.02	−0.97±0.45	0.03	1.35±0.55	0.02	1.200.49	0.03	−4.22±0.56	0.31	4.31±0.74	0.63
Later(IIII+IV)	−13.82±0.56		−2.17±0.29		−0.19±0.33		−0.18±0.36		−5.04±0.49		3.78±0.89	

## Discussion

MiRNA microarray studies provide amounts of information, but a common drawback is the lack of consistency among different studies. According to the reports of Griffith et al. and Chan et al. [16], [17], a logical solution to this problem would be to determine the consistency between different studies used different microarray platforms. Several systematic reviews [15]–[17] have used this method to determine differentially expressed genes or miRNAs in various disease states. Applying a similar method, we observed that a total of 48 differentially expressed miRNAs were reported in at least two independent studies among eight GC miRNA profiling studies [7]–[14]. Among these, 39 miRNAs were reported to be altered in a consistent direction, while the findings for nine were inconsistent. Among the 39 miRNAs that had consistent changes, 20 miRNAs were consistently upregulated in GC compared with noncancerous gastric tissue, and 19 were consistently downregulated in GC. We identified the five miRNAs that were most consistently upregulated (miR-21, miR-106a, miR-17, miR-18a and miR-20a) and two most consistently downregulated (miR-378 and miR-638) in at least four profiling studies. Then, we validated these findings using real-time PCR, which further supported the findings of this systematic review. We also determined that the expression of these miRNAs correlated with the clinicopathological features of GC, which suggested that these miRNAs may be useful as biomarkers for GC.

One of the most consistently reported upregulated miRNA in our systematic review was miR-21, which has altered expression and oncogenic activity in different human cancers. Cui et al. [18] showed that the expression of miR-21 was significantly higher in GC tissue compared with adjacent normal tissue. The expression of miR-21 has also been found to be higher in patients with GC with lymph node metastasis than those without, and was also significantly correlated with the histological tumor type and pTNM stage [19], which was validated by our study. Moreover, higher expression levels of miR-21 predicted poor survival in patients with GC [19]. Other studies have found that miR-21 may promote tumor proliferation and invasion in GC by suppressing the expression of PTEN or PDCD4 [20], [21]. Additionally, previous studies have also revealed oncogenic activity of miR-21 in colorectal cancer [22], breast cancer [23] and esophageal cancer [24].

MiR-106b was also consistently reported as an upregulated miRNA in GC tissue by this and previous studies [14], [25]. The high expression of miR-106b has been previously associated with lymph node metastasis [25], [26], and this was validated in our study. Kim et al. [27] found that miR-106b may exert its oncogenic activity by suppressing p21 expression in GC. MiR-106b could induce epithelial-to-mesenchymal transition (EMT) and a tumor initiating cell phenotype in breast cancer by targeting Smad7 and Six1 and activating TGF-β signaling [28]. It may also promote cell proliferation in human hepatocellular carcinoma by downregulating the expression of APC [29].

MiR-17 has known oncogenic activity in humans, and was found to be upregulated in 77.2% of tissue samples of GC compared with adjacent normal gastric tissue. It promotes cell cycle progression and inhibit apoptosis in GC by targeting p21 and p53INP1 (tumor protein p53-induced nuclear protein 1) [30]. Circulating levels of miR-17 are elevated in GC patients, and the concentration of miR-17 is significantly associated with the TNM stage and grade of GC [31]. However, our study found that the expression of miR-17a was higher in the cases of GC without lymph node metastasis than those with lymph node involvement, which may have been a consequence of the small sample size in our study. The high expression of miR-17 is significantly correlated with poor survival outcomes [31]. Previous studies have also found that miR-17 has oncogenic activity in colorectal cancer [32], breast cancer [33] and pancreatic cancer [34].

MiR-18a was found to be upregulated in four studies in this systematic review, and is known to have oncogenic activity in humans. Wu et al. [35] revealed that the expression of miR-18a was significantly upregulated in GC tissue compared with normal gastric tissue, and could directly target PIAS3 (protein inhibitor of activated signal transducer and activator of transcription 3) and was positively correlated with levels of Survivin, Bcl-xl and c-myc. Moreover, the upregulation of miR-18a has been reported in nasopharyngeal carcinoma [36], pancreatic cancer [37], hepatocellular carcinoma [38] and breast cancer [39].

Mir-20a is another miRNA with oncogenic activity, and was found to be upregulated in four studies in this literature. It has been demonstrated that the circulating level of miR-20a is significantly elevated in GC patients compared to healthy controls, and this is significantly associated with the stage and grade of the tumor [31], [40]. Our study also found that miR-20a was significantly elevated in GC tissues and was significantly associated with lymph node metastasis. Moreover, the upregulation of miR-20a has previously been found in cervical cancer, prostate cancer and ovarian cancer, and this miRNA could promote the cell proliferation or invasion of these cancers [41]–[43].

The most consistently downregulated miRNA in this systematic review was miR-378, which was found to be downregulated in five studies. MiR-378 has been demonstrated to have anti-oncogenic activity in humans [44]. The exogenous expression of miR-378 markedly suppresses the proliferation of GC cells by suppressing CDK6 and VEGF signaling [44]. In our study, although we found that the expression of miR-378 was downregulated in GC tissues, no relationship was found between the expression of miR-378 and the clinicopathological features of GC. This may have been due to the small sample size of this study. It is also reported that miR-378 is significantly downregulated in colorectal cancer, and may play an important tumor suppressor role in this cancer [45]. However, other studies have found that miR-378 may have oncogenic activity in other cancer types [46]–[49]. Therefore, the exact role of miR-378 in carcinogenesis needs to be further elucidated.

Furthermore, we also found that some of the candidate miRNAs identified in our study were slso identified as serum biomarkers in various cancers. For example, serum miR-21 was significantly elevated in perioperative serum from adenomas and colorectal cancer (CRC), and was an independent prognostic marker for CRC [50], [51]; Plasma miR-106b, together with miR-20a and miR-221 have the potential as novel biomarkers for early detection of gastric cancer [40]; Circulating miR-17 may used as a novel noninvasive biomarker for nasopharyngeal carcinoma [52], gastric cancer [53] and CRC [54]; Serum miR-18a may be used as a novel biomarker in breast cancer [55], colorectal cancer [56], hepatocellular carcinoma [57], and pancreatic cancer [58]; Circulating miR-378 may be used as a biomarker in renal cell carcinoma [59] and gastric cancer [60]. These studies further confirmed the importance of the indentified miRNAs, and may expand the application scope of these miRNAs.

In conclusion, our systemic review identified five upregulated miRNAs (miR-21, miR-106b, miR-17, miR-18a and miR-20a) and one downregulated miRNA (miR-378) that are potential novel biomarkers for GC. These miRNAs have been shown to have diagnostic and/or prognostic potential for this cancer and warrant further investigation. Further studies that focus on these miRNAs will help to determine a panel of diagnostic and prognostic GC biomarkers with appropriate levels of sensitivity and specificity.

## Supporting Information

Table S1 Differentially expressed miRNAs identified in each included study.
(XLS)

Table S2 Raw Ct value of the selected miRNAs.
(XLS)

Table S3 PRISMA Checklist.
(DOC)
